# Risk factors for tuberculosis: A case–control study in Addis Ababa, Ethiopia

**DOI:** 10.1371/journal.pone.0214235

**Published:** 2019-04-02

**Authors:** Ezra Shimeles, Fikre Enquselassie, Abraham Aseffa, Melaku Tilahun, Alemayehu Mekonen, Getachew Wondimagegn, Tsegaye Hailu

**Affiliations:** 1 Armauer Hanson Research Institute, ALERT Compound, Addis Ababa, Ethiopia; 2 School of Public health, College of Health Sciences, Addis Ababa University, Black Lion Hospital, Addis Ababa, Ethiopia; 3 Ethiopian Public Health Association, Kirkos Sub City, Addis Ababa, Ethiopia; 4 KNCV Tuberculosis Foundation, Addis Ababa, Ethiopia; Department of Internal Medicine, Federal Teaching Hospital Abakaliki, Ebonyi State, Nigeria, NIGERIA

## Abstract

**Background:**

Tuberculosis remains a major public-health problem in the world, despite several efforts to improve case identification and treatment compliance. It is well known cause of ill-health among millions of people each year and ranks as the second leading cause of death from infectious disease worldwide. Despite implementation of the World health organization recommended strategy, the reductions in the incidence of TB have been minimal in high burden countries.

**Objectives and methods:**

A case control study was carried out to assess the risk factors of TB, where cases were newly registered bacteriologically confirmed pulmonary TB patients with age greater than 15 years who present at twenty health centres in Addis Ababa. Controls were age and sex matched attendees who presented in the same health centers for non-TB health problems.

**Results:**

A total of 260 cases and 260 controls were enrolled in the study and 45.8% of cases and 46.2% of controls were in the 26–45 years age bracket. According to the multivariable logistic regression analysis, seven variables were found to be independent predictors for the occurrence of TB after controlling possible confounders. Patients who live in house with no window or one window were almost two times more likely to develop tuberculosis compared to people whose house has multiple windows (AOR = 1.81; 95% CI:1.06, 3.07). Previous history of hospital admission was found to pose risk almost more than three times (AOR = 3.39; 95% CI: 1.64–7.03). Having a household member who had TB was shown to increase risk of developing TB by three fold (AOR = 3.00; 95% CI: 1.60, 5.62). The study showed that illiterate TB patients were found to be more than twice more likely to develop TB compared to subjects who can atleast read and write (AOR, 95% CI = 2.15, 1.05, 4.40). Patients with household income of less than 1000 birrs per month were more than two times more likely to develop TB compared to those who had higher income (AOR = 2.2; 95% CI: 1.28, 3.78). Smoking has also been identified as important risk factor for developing TB by four times (AOR = 4.43; 95% CI: 2.10, 9.3). BCG was found to be protective against TB reducing the risk by one-third (AOR = 0.34; 95% CI: 0.22, 0.54).

**Conclusion:**

This study showed that TB is more common among the most agile and economically active age group, and number of windows, history of hospital admission, a household member who had TB, illiteracy, low household income and smoking and lack of BCG scar were identified as independent risk factors. Therefore it is imperative that the TB control effort need a strategy to address socio economic issues such as poverty, overcrowding, smoking, and infection control at health care facilities level is an important intervention to prevent transmission of TB within the facilities.

## Introduction

Tuberculosis (TB) remains a major public-health problem in the world, despite several efforts to improve case identification and treatment compliance. It is the single highest curable infectious killer in today’s world.

It is well known cause of ill-health among millions of people each year and ranks as the second leading cause of death from infectious disease worldwide, after the human immunodeficiency virus (HIV). According to World Health Organization (WHO) latest estimate, 10.4 million people fell ill with TB in 2016 and 1.6 million died from the disease [1].

Tuberculosis usually affects the lungs and it is transmitted from person to person via droplets, and over 90% of people infected with the tubercle bacillus will not develop TB disease [2].

Those at risk of developing the disease following infection with the tubercle bacillus include poorly nourished individuals, and those with poor immune defenses such as persons infected with HIV, diabetics, alcoholics, patients with leukemia and patients receiving immunosuppressive therapies [3].

Despite implementation of the WHO recommended directly observed therapy short course (DOTS)TB control strategy, the reductions in the incidence of TB have been minimal in high burden countries [1]. Ethiopia is one of the 30 high TB, TB/HIV and RR/MDR-TB burden countries in the world [4] and total estimated incidence of TB in 2016 stands at 177 per 100,000 populations [1]. This is relatively lower than the previous years of 199 per 100,000 and 207 per 100,000 which were reported in 2015 and 2014 respectively [5, 6].

Globally TB incidence is falling at about 2% per year by 2020, these figure need to improve to 4–5% per year, to reach the first (2020) milestones of the End TB Strategy [1].

Because of this slow decline of TB incidence there is currently renewed interest in finding new TB control strategies. Focus has been on such strategies as adding to the current TB drugs, finding a TB vaccine and designing shorter TB regimens. However, knowledge of what makes some persons develop TB and others not (risk factors) has potential of helping further to refocus the search for novel public health TB control strategies. Reported TB risk factors include HIV infection, male sex, co-morbidities such as diabetes, and family history of TB, absence of a Bacillus Calmette–Guérin (BCG) scar, smoking, alcohol use, single marital status, overcrowding, and poor socioeconomic status [7–10].

With rising trend of TB, affecting mainly developing countries, there is a need to re-examine the characteristics of the patients and understanding the contributing factors, in order to adjust and adapt TB control policies. National TB program of Ethiopia has supported a sustained public education campaign through the media on the symptoms of TB, mode of transmission, the importance of seeking care, risk of MDR-TB and the fact that TB is curable. To further step up the fight, the government has also put a lot of effort in ensuring adequate availability of drugs and adequately trained staff in all government and selected mission hospitals. However, its current effort to find, treat and cure everyone who gets ill with the disease is not sufficient.

Therefore, the study aimed at establishing the risk factors contributing to the development of TB. The findings of the study will help for policy improvement and planning for successful tuberculosis control in Ethiopia.

## Materials and methods

### Study population

A case control study was carried out to assess the risk factors of TB and **cases** were newly registered bacteriologically confirmed pulmonary TB patients with age greater than 15 years who present at twenty health centres in Addis Ababa that are selected for the study in consultation with Addis Ababa Health Bureau, while controls were age and sex matched attendees who presented in the same health centers for non-TB health problems.

National TB programme of Ethiopia notified 127,407 TB cases in 2016, of which 1,571 were previously treated cases. Among them 68% were pulmonary, and of which 55% bacteriologically confirmed. About half (45%) of these cases were females. Treatment coverage rate was 69% and great majority (81%) knew their HIV status, of whom 8% were found to be positive [1].

### Sample size

The sample size determined for the study was calculated using the formula when the interest is to test a hypothesis comparing some exposure of two groups, seen in Box 1.

**Box 1. Sample size calculation for the study.**

$$\text{N}=\frac{{\left[{\text{Z}}_{\alpha }{\text{P}}_{1}\left(1-{\text{P}}_{1}\right)+{\text{Z}}_{\beta }{\text{P}}_{2}\left(1-{\text{P}}_{2}\right)\right]}^{2}}{{\left({\text{P}}_{1}-{\text{P}}_{2}\right)}^{2}}$$

**P**_**1**_ is the likely percentage of exposure on cases

**P**_**2**_ is the likely percentage of exposure on controls

**α** the significance level

**1-β** the power of the test

Considering HIV as most important risk factor for TB disease, P_**1**_ which is the risk of TB disease among those infected with HIV was determined based on the latest data from Ministry of health on national report on TB/HIV collaborative activities, which showed that 20% of TB patients were HIV positive [11]. Similarly P_**2**_ was determined based on the adult prevalence of HIV in Addis Ababa reported on the single point estimate, which was 8.5%. With 90% Power and 95% confidence interval the total sample size was calculated to be 236. In consideration of 10% non-response rate the sample size was adjusted to be 260. As the ratio of cases to controls was decided to be one to one, similar number of controls are also planned to be included in the study which makes the total sample size 520.

- P_**1**_ (risk factor among exposed) = Proportion of HIV among TB patients = 20% (MOH, 2015/16)
- P_**2**_ (risk factor among non-exposed) = Proportion of HIV among controls (non-TB), ie the prevalence of adult HIV prevalence in Addis Ababa, 8.5, based on the single point estimate (2015/16)
- Power = 90%
- Confidence interval = 95%
- Ratio of cases and control = 1:1
- Calculated sample size = 236

### Sampling procedure

1. *Selection of cases*: The cases were newly detected bacteriologically confirmed pulmonary TB patients aged >15 years, enrolled for treatment in the selected health centres in Addis Ababa. The data collection has taken place by including all newly registered TB patients until the required sample size was reached.
2. *Selection of health facility control*; OPD attendee in the same facility with age (within 5 year age bands) and sex-matched with a respective case were selected as control. Attendees fitting the age and sex criteria were approached on a clinic day. *If he/she refused to be involved in the study*, *the next eligible clinic attendee was approached*. The controls were seen by the study doctor to manage their medical complaint and to have a clinical screening to exclude pulmonary TB. If the client had any sign suggestive of TB, the necessary laboratory tests for TB were ordered according to the national diagnostic algorithm. Since AFB microscopy tests is provided free of charge, there was no cost incurred. However if there is a need for other investigations like X-ray, the cost was covered by the study.

The inclusion and exclusion criteria are summarized in seen in Box 2.

**Box 2. Inclusion and exclusion criteria for the study**

**Inclusion criteria**

**Cases;**
- Bacteriologically confirmed pulmonary TB patients enrolled for treatment in selected health centres in Addis Ababa.
- Age more than 15 years

**Controls;**
- Attendees of health facility for non-TB health problems
- Age more than 15 years
- Sex and age matched with case (within 5 year age bands)

**Exclusion criteria**

- Smear negatives TB, relapse, Treatment failure, Treatment after lost to follow up.
- All health facility controls with clinical sign or symptom suggestive of TB

### Data analysis

The data captured in the questionnaires was grouped and examined for errors, then cleaned, and entered into RedCap version 8.03. Data was analysed using STATA version 11 statistical software. Descriptive statistics was used to summarize patients’ socio-demographic, living condition, life style, previous medical history and knowledge of TB. Association between variables were determined using odds ratio and 95% CI. Multivariate analysis was run by selecting those variables that appeared to have a P-value of < 0.05 in the bivariate analysis to control the confounding effect of different variables while assessing the effect of each variable on the likelihood of TB development. P-value of < 0.05 was considered as statistical significance.

### Ethical considerations

The research proposal was subjected to screening for scientific and ethical integrity by Ethical review committee at School of Public Health, and institutional review board of the College of Health Sciences, Addis Ababa University. Further screening was done by institutional review board of ALERT/AHRI, as well as Addis Ababa health bureau. A support obtained from these organizations gave a green light to carry out the study. The management of each of the selected health facilities were given orientation to ensure their support and facilitation of the data collection process.

Permission and consent to participate in the study was obtained from every individual respondent and patient‘s disease status were kept confidential.

### Time frame

The study commenced in January 2017 and the data was collected in the period till 30 December 2017.

## Results

### Socio- demographic characteristics of the participants

A total of 260 cases and 260 controls participated in the study. The median age for cases was 27 (range 22–35) years, and it was also 27 (range 23–34) years for control group, and the majority (45.8%]) of cases and 46.2% of controls were within the age group of 26 and 45 years, while 43.1% of cases and 44.2% of controls were under 25 years. More than half (53.3%) of the cases and controls were males while 46.7%, as seen in Table 1.

**Table 1:** Socio-demographic characteristics of TB patients and controls, Addis Ababa, December 2017 (n = 260).

Characteristics	Cases (n = 260)		Controls (n = 260)	
	Frequency	Percent	Frequency	Percent
**Sex**				
Male	145	55.8	145	55.8
Female	115	44.2	115	44.2
**Age category (Years)**				
≤ 25	112	43.1	115	44.2
26–45	119	45.8	120	46.2
> 45	29	11.2	25	9.6
**Marital status**				
Single	135	51.9	121	46.5
Married	98	35.7	131	50.4
Divorced	18	6.9	6	2.3
Widowed	9	3.5	2	0.8
**Educational status**				
Illiterate	41	15.8	18	6.9
Read and write	21	8.1	22	8.5
Up to elementary	86	33.1	61	23.5
Secondary school	83	31.9	95	36.5
College or more	29	11.2	34	24.6
**Occupation**				
Employed/merchants/wage	184	67.7	150	57.7
Unemployed	76	32.3	110	42.3

**Cases**: median age 27 years (Interquartile range: 23–36), and mean age 30 years (standard deviation ±11.27),

**Controls**: median age 27 years (range 23–35), and mean age 30 years (standard deviation ±10.89)

About fifty two percent of the cases were single and 35.7% married, while among controls 46.5% of were single and 50.45 were married. About three fourth of the cases (76.2%) and 84.5% of controls had attained at least primary level of education and, about two-third (67.7%) of the cases and 57.7% of the controls reported to have formal employment or merchants or daily wage.

### Living conditions

The median family size was 3 for cases and 4 for controls, and more than half (53.1%) of the cases have less than four family members, while 44.6% of the controls have less than four family members. More than a fifth (21.9%) of cases and 21.5% of controls reported history of living in congregate settings. Most of the participants, which include 42.3% of cases and 57.3% of the controls, live in households with monthly income of more than 2000 birrs, while 29.6% of cases and 15.4% of controls live in household with less than 1000 Birrs per month. More than half of the cases (53.8%) and 38.1% of the controls live in single room house, and 22.7% of cases and 35.8% of controls live in two room house. In terms of number of windows, 69.6% of cases and 38.1% of controls live in houses with single window or no window, while 21.2% of cases and 36.2% of controls in houses with two to three windows. Close to half (48.7%) of cases and 33.2% of controls claimed opening their windows the whole day, while 18.1% of cases and 13.5% of controls never open it. Almost one-sixth (16.5%) of the cases and 7.3% of controls had household members who had TB, as seen in Table 2.

**Table 2:** Living conditions of TB patients and controls, Addis Ababa, December 2017 (n = 260).

Characteristics	Cases (n = 260)		Controls (n = 260)	
	Frequency	Percent	Frequency	Percent
**Size of the family**				
<4	138	53.1	116	44.6
4 to 6	70	26.9	103	39.6
>6	52	20.0	41	15.8
**Ever lived in congregate settings**				
Yes	57	21.9	56	21.5
No	203	78.1	204	78.5
**Average household monthly income (ETB)**				
<1000 birrs	77	29.6	40	15.4
1000–2000 birrs	73	28.1	71	27.3
>2000 birrs	110	42.3	149	57.3
**Number of rooms**				
One	140	53.8	99	38.1
Two	59	22.7	93	35.8
Three or more	61	23.5	68	26.2
**Number of windows**				
0–1	181	69.6	145	55.8
2–3	55	21.2	94	36.2
>3	24	9.2	21	8.1
**Duration the window in the house remain open in a day**				
Whole day	94	48.7	76	33.2
Half day	38	19.7	74	32.3
2–3 hours	26	13.5	48	21.0
Never	35	18.1	31	13.5
**Members of household with TB**				
Yes	43	16.5	19	7.3
No	217	83.5	241	92.7

### Life style

As seen in Table 3, some (16.2%) of the cases and 5.8% of controls smoke cigarettes, and 40.5% of the cases smoke 6 to 10 cigarettes per day while 53.3% of controls do the same. About a third (33.5%) of cases and 30.8% of controls reported alcohol consumption, of whom and, majority of both groups, 47.1% of cases and 58.7% of controls, do it rarely.

**Table 3:** Personal life style of TB patients and controls, Addis Ababa, December 2017 (n = 260).

Characteristics	Cases (n = 260)		Controls (n = 260)	
	Frequency	Percent	Frequency	Percent
**Cigarette smoking**				
Yes	42	16.2	15	5.8
No	218	83.8	245	94.2
**Number of cigarettes per day**				
Less than 5	13	31.0	7	46.7
6–10	17	40.5	8	53.3
10–20	5	11.9		
More than 20	7	16.7		
**Alcohol consumption**				
Yes	87	33.5	80	30.8
No	173	66.5	180	69.2
**Frequency of drinking**				
Daily	18	20.7	8	10
Twice per week	18	20.7	12	15
Weekly	5	5.7	8	10
Monthly	5	5.7	5	6.3
Rarely	41	47.1	47	58.7

### Previous medical history

Table 4 shows that about a quarter of the cases (25.4%) and close to half of the controls (47.7%) had BCG vaccination. Almost three fourth of both cases and controls had history of visiting a health facility during the 12 months before diagnosis of current illness, and majority did it two to five times, which include 61.8% and 52.3% of cases and of controls respectively. While 14.2% of cases reported history of hospital admission of different duration, 6.2% of controls had similar experience. Small portion of both cases and controls (14.6%) had some kind of chronic illness.

**Table 4:** Past medical history of TB patients and controls, Addis Ababa, December 2017 (n = 260).

Characteristics	Cases (n = 260)		Controls (n = 260)	
	Frequency	Percent	Frequency	Percent
**BCG vaccination**				
Yes	66	25.4	124	47.7
No	194	74.6	136	52.3
**History of visiting health facility**				
Yes	191	73.5	193	74.2
No	69	26.5	67	25.8
**Frequency of visit**				
Once	47	24.6	66	34.2
Two to five times	118	61.8	101	52.3
More than five times	26	13.6	26	13.5
**History of hospital admission**				
Yes	37	14.2	16	6.2
No	223	85.8	244	93.8
**History of chronic illness**				
Yes	38	14.6	38	14.6
No	222	85.4	222	85.4

### Knowledge about tuberculosis

As seen in Table 5, majority of cases (83.8%) and controls (90.8%) have heard of TB and many of them mentioned cough as main symptom. Regarding route of transmission of TB, majority of cases (83.1%) and 88.5% of controls mentioned airborne transmission, and in terms of curability most of the cases (86.5%) and 90.4% of controls mentioned that TB is curable.

**Table 5:** Knowledge of TB patients and controls, Addis Ababa, December 2017 (n = 260).

Characteristics	Cases (n = 260)		Controls (n = 260)	
	Frequency	Percent	Frequency	Percent
**Have you heard of a disease called Tuberculosis?**				
Yes	218	83.8	236	90.8
No	42	16.2	24	9.2
**What are the symptoms of Tuberculosis?**				
Cough	209	80.4	223	85.8
Weakness	112	43.1	128	49.2
Sputum	97	37.3	73	28.1
Weight loss	95	36.5	85	32.7
Haemoptysis	92	35.4	57	21.9
Fever	40	15.4	62	23.8
**How is TB transmitted from person to person**				
Through breathing	216	83.1	230	88.5
Other routes/ Do not know	44	16.9	30	11.5
**Is TB a curable disease**				
Yes	225	86.5	235	90.4
No	35	13.5	25	9.6

### Comparative analysis

The bivariate analysis revealed a crude association with developing TB, for illiterate, household size of less than 4 people, household income less than 1000 birrs, those living in single room house, with no or one window, smoking, BCG scar and history of hospital admission. All other variables didn’t show any significant association, as presented in Table 6.

**Table 6:** Bivariate analysis of risk factors among TB patients and controls, in Addis Ababa, December, 2017: Cases = 260, Controls = 260.

Characteristics	Cases (n = 260)		Controls (n = 260)		
	N	%	N	%	COR(95% CI)
**Age category (Years)**					
≤ 25	112	43.1	115	44.2	Reference
26–45	119	45.8	120	46.2	1.05(0.73, 1.51)
> 45	29	11.2	25	9.6	1.11(0.61, 2.04)
**Marital status**					
Unmarried	162	62.3	129	49.6	1.24(0.87, 1.75)
Married	98	35.7	131	50.4	Reference
Educational status*					
Illiterate	41	15.8	18	6.9	**2.51(1.40, 4.51)**
Literate	219	84.3	242	93.1	Reference
Size of the family*					
<4	138	53.1	116	44.6	**1.75(1.18, 2.58)**
4–6	70	26.9	103	39.6	Reference
>6	52	20.0	41	15.8	1.86(1.12, 3.10)
Average household monthly income*					
<1000 birrs	77	29.6	40	15.4	**2.60(1.65, 4.10)**
1000–2000 birrs	73	28.1	71	27.3	1.39(0.92, 2.09)
>2000 birrs	110	42.3	149	57.3	Reference
Number of rooms*					
One	140	53.8	99	38.1	**2.29(1.47, 3.37)**
Two	59	22.7	93	35.8	Reference
Three or more	61	23.5	68	26.2	1.41(0.87, 2.27)
Number of windows*					
0–1	181	69.6	145	55.8	**2.13(1.43, 3.17)**
2–3	55	21.2	94	36.2	Reference
>3	24	9.2	21	8.1	1.95(0.99, 3.83)
Members of household with TB*					
Yes	43	16.5	19	7.3	**2.51(1.42, 4.44)**
No	217	83.5	241	92.7	Reference
Smoking*					
Yes	42	16.2	15	5.8	**3.14(1.69, 5.83)**
No	218	83.8	245	94.2	Reference
**Alcohol**					
Yes	87	33.5	80	30.8	Reference
No	173	66.5	180	69.2	1.13(0.61, 1.27)
BCG scar*					
Yes	66	25.4	124	47.7	**0.37(0.26, 0.54)**
No	194	74.6	136	52.3	Reference
**History of visiting health facility in the past 12 months**					
Yes	191	73.5	193	74.2	0.96(0.6, 1.53)
No	69	26.5	67	25.8	Reference
History of hospital admission*					
Yes	37	14.2	16	6.2	**2.53 (1.36, 4.67)**
No	223	85.8	244	93.8	Reference
**Knowledge on TB: Is TB curable?**					
Yes	225	86.5	235	90.4	1.46(0.87, 2.59)
No	35	13.5	25	9.6	Reference

Notes:

*Variables that showed significant association during bivariate analysis at p<0.05.

**Abbreviations**: **TB**: tuberculosis; **COR**: crude odds ratio, **CI**: confidence interval.

### The overall predictors of developing TB

All variables which had shown statistically significant association during the bivariate analysis, were collectively entered in the multivariable analysis, and according to the multivariable logistic regression analysis, seven variables were found to be independent predictors for the occurrence of TB after controlling possible confounders. Illiterate patients were found to be more than twice more likely to develop TB compared to subjects who can atleast read and write in local language (adjusted odds ratio [AOR] = 2.15, with 95% confidence interval [CI]: 1.05, 4.40). Patients with household income of less than 1000 birrs per month were more than two times more likely to develop TB compared to those who had higher income (AOR = 2.2; 95% CI: 1.28, 3.78). Those patients living in house with no window or one window were almost two times more likely to develop TB compared to people whose house has multiple windows (AOR = 1.81; 95% CI:1.06, 3.07).

Having a household member who had TB was shown to increase risk of developing TB by three fold (AOR = 3.00; 95% CI: 1.60, 5.62). Similarly smoking was found to be important risk factor for developing TB by four times (AOR = 4.43; 95% CI: 2.10, 9.3). BCG was found to be protective against TB reducing the risk by one-third (AOR = 0.34; 95% CI: 0.22, 0.54). History of hospital admission was found to increase risk by more than three times (AOR = 3.39; 95% CI: 1.64, 7.03), as seen in Table 7.

**Table 7:** Multivariate analysis of risk factors among the study participants TB patients and controls, at health centres in Addis Ababa, 2017: Cases = 260, Controls = 260.

Characteristics	Cases (n)	Control (n)	COR (95% CI)	AOR (95% CI)	P-value
**Educational status**					
Illiterate	41	18	**2.51(1.40, 4.51)**	**2.15(1.05, 4.40)**	0.034*
Literate	219	242	Reference		
Size of the family*					
<4	138	116	**1.75(1.18, 2.58)**	1.51(0.92, 2.46)	0.098
4–6	70	103	Reference		
>6	52	41	1.86(1.12, 3.10)	1.54(0.84, 2.79)	0.154
Average household monthly income*					
<1000 birrs	77	40	**2.60(1.65, 4.10)**	**2.20(1.28, 3.78)**	0.004*
1000–2000 birrs	73	71	1.39(0.92, 2.09)	1.30(0.8, 2.10)	0.281
>2000 birrs	110	149	Reference		
Number of rooms**					
One	140	99	**2.29(1.47, 3.37)**	1.29(0.76, 2.18)	0.332
Two	59	93	Reference		
Three or more	61	68	1.41(0.87, 2.27)	1.56(0.83, 2.93)	0.160
Number of windows*					
0–1	181	145	**2.13(1.43, 3.17)**	**1.81(1.06, 3.07)**	0.028*
2–3	55	94	Reference		
>3	24	21	1.95(0.99, 3.83)	2.13(0.94, 4.79)	0.067
Members of household with TB*					
Yes	43	19	**2.51(1.42, 4.44)**	**3.00(1.60, 5.62)**	0.001**
No	217	241	Reference		
Smoking*					
Yes	42	15	**3.15(1.69, 5.83)**	**4.43(2.10, 9.3)**	<0.001*
No	218	245	Reference		
BCG scar*					
Yes	66	124	**0.37(0.26, 0.54)**	**0.34(0.22,0.54)**	<0.001*
No	194	136	Reference		
History of hospital admission*					
Yes	37	16	**2.53 (1.36, 4.67)**	**3.39(1.64,7.03)**	0.001**
No	223	244	Reference		

Notes:

*Variables that showed significant association during bivariate analysis at p<0.05.

**Abbreviations**: **TB**: tuberculosis; **COR**: crude odds ratio, **AOR**: Adjusted odds ratio, **CI**: confidence interval.

## Discussion

A total of 520 patients participated in this case control study which was carried out in Addis Ababa, Ethiopia, which include 260 bacteriologically confirmed pulmonary TB patients and 260 clinic attendees. There is a high burden of Tuberculosis in many countries which may be attributed to its demographic and socio-economic profile like poverty, lack of knowledge, attitude and practice, overcrowding, malnutrition, co-morbidity, etc. Adequate information on epidemiological factors is essential in formulating national policy and to redirect health resources in order to control the transmission of TB as well as ensure better patient management. As no single factor is fully attributable for emergence of TB, and there is information gap on factors contributing to occurrence of TB, this study has tried to explore different socio-demographic factors. Ethiopia is among the 30 high burden countries for TB, TB/HIV as well as drug resistant TB that collectively contribute about 85–89% of the global burden [4].

The socio-demographic characteristics of the study participants indicated that 45.8% of cases and 46.2% of controls were in the 26–45 years age bracket, which is the most agile and economically active age group. This is similar to another study among TB patient in North Ethiopia, Gondar and Borumeda, which showed (54.9%) of the cases were within the age ranges of 26–45 years [12]. Whereas other study at St Peter hospital, Addis Ababa, showed that 29.9% were in the age bracket 26–45 [13]. Generally, the findings is consistent with other studies, which reported a rapid rise in TB morbidity and mortality among this young adult population mostly between 15–44 years of age [14]. High risk of infection in this age group relates to having a higher number of social contacts in the community during young adulthood [15].

The study revealed, majority (55.8%) were males and (44.2%) were females which was the same for both cases and controls as the study applied one to one match by sex and age group. Similar finding was observed in other studies where 60.5% (15), 57.5% [12] of participants were males. Some studies revealed lower percentage of male patients (43.3%) [16], however most studies reported very high percentage of male: (70.0) [17], 70.7% [18] and 71.1% [19]. The male: female ratio globally was reported to be consistently high, over last three years; 1.7 [6], 1.6 [5] 1, 1.7 [1] respectively. Male populations are vulnerable for TB as they have more chance to contact with the carriers due to their outdoor social activities.

Majority of the cases (53.1%) have less than four family members, which is higher than the controls (44.6%). Overall household’s monthly income was reported to be lower for cases compared to controls, where 29.6% and 15.4% of cases and controls respectively live in household with less than 1000 Birrs per month. Other similar studies showed average monthly family income of upto 1000 birrs among 86.9% of participants in rural setting [20] and 72.6% in urban area [21]. Housing conditions were poorer for cases compared to controls, as higher percentage of cases (53.8%) live in single room house that controls (38.1%), whereas more controls (35.8%) than cases (22.7%) live in a two room house. Number of windows per house was also much lower for cases than controls, for instance 69.6% of cases and 55.8% of controls live in houses with single window or no window, while the figure is contrary to this when it comes to living in houses with multiple windows. In developing countries like Ethiopia most of the poor families face economic constrains, which leads to malnutrition, poverty, overcrowding, poor hygiene, decreased health care seeking attitude forming a vicious cycle of agent-host- environment increasing the risk for communicable diseases such as TB. Overcrowding has been previously documented as a strong risk for TB [22].

Almost fifth (18.1%) of the cases and 7.3% of controls had household members who had TB, which is close to findings of other study where 17.5% of TB patients reported family history of TB [16]. Close contact is well described risk factor for TB [23–25]. Close contact was estimated to account for 9–13% of the TB cases in Malawi [26]. In a systematic review by Fox GJ et al. the prevalence of active TB and latent TB infection among TB contacts was 3.1% and 51.5% respectively [27]. The effect of former experience of TB within the household has been observed consistently in other studies and such effect increased with the number of persons who had TB in the past [28]. Furthermore, there was some evidence that this effect was higher when the former TB case was in close family link with the index TB case, as compared with unrelated household members. As reported in another study, within the households of TB case, the risk of TB infection increased with social proximity to the case, and that effect was persistently higher in first-degree than in more distant relatives [8].

Some (16.2%) of the cases and 6.5% of controls smoke cigarettes, and 40.5% of the cases smoke 6 to 10 cigarettes per day while 52.9% of controls do the same. The association between tobacco smoking and TB is well described in other studies [29–31]. The prevalence reported in this study does not differ significantly from those reported in other studies in Africa [32] but differs from those in other continents [33]. Such differences could be attributed to background smoking prevalence in the general population.

This study also investigated alcohol use among TB patients and controls. About a third (33.5%) of cases and 30.8% of controls reported alcohol consumption, of whom 36.2% drink rarely, majority of both groups do it rarely. The prevalence of alcohol consumption in this study is lower than the result of national survey conducted in the general population where 35% of women and about half of men (46%) reported drinking alcohol [34]. Practically there is no major difference in alcohol consumption among cases and controls.

Almost three fourth of both cases and controls had history of visiting a health facility during the 12 months before diagnosis of current illness. History of hospital admission was more than double among cases compared to controls. Chronic illness such as diabetes mellitus reduce the competency of the immune system, pulmonary diseases minimize function of the cilia and removal of inhaled substances, and hence increases risk of TB. Besides, people with chronic illness visit health facilities frequently and have increased risk of acquiring TB.

According to the multivariable logistic regression analysis, seven variables were found to be independent predictors for the occurrence of TB after controlling possible confounders.

Those subjects who live in house with no window or one window were almost two times more likely to develop TB compared to people whose house has multiple windows (AOR = 1.81; 95% CI:1.06, 3.07). Inadequate ventilation and overcrowding has been documented as a risk factor for TB from several other studies in a variety of settings [35, 36]. People who live in house without any window or single window at high risk of TB as it compromises ventilation and increase risk of transmission of airborne infection such as TB. Besides, they might be among the lowest income groups in the society, and if have bigger family size, that leads to overcrowding and malnutrition which will further increase the risk. This hence increase vulnerability to contagious diseases, and that creates favourable environment for TB transmission.

Patients who had previous history of hospital admission were more than three times more likely to develop TB compared to those who had no previous history of hospital admission (AOR = 3.39; 95% CI: 1.64–7.03). This indicates that visiting health facilities has risk of acquiring TB, demonstrating the need to establish a strong system for infection control in health care settings. A longitudinal study conducted in Western Ethiopia revealed that the incidence rate of hospital acquired infection was 28.15 [95% C.I:24.40, 32.30] per 1000 patient days while the overall prevalence was 19.41% (95% C.I: (16.97–21.85), and pneumonia and other respiratory tract infections were among the top ten diseases [37]. Similarly another study in Northern Ethiopia showed a mean prevalence of hospital acquired infection of 14.9% (95% confidence interval 12.7–17.1), Pneumonia and respiratory tract infection being the second commonest with prevalence of 18.5% (95% confidence interval of 11.9–25.9) [38]. Therefore, transmission of infection in hospital setting is common in Ethiopia and people who have history of admission have high chance of acquiring infection during their stay in the ward. The risk is higher among people who had longer duration of admission and among those with repeated admission.

Having a household member who had TB was shown to increase risk of developing TB by three fold (AOR = 3.00; 95% CI: 1.60, 5.62). Another study in Ethiopia reported similar findings where contact with active tuberculosis patient higher risk (AOR = 5.90; 95% CI = 2.30–15.30) [39], and similarly in Uganda, household TB contact in last 2 years with APR 1.91 (95% CI 1.55–2.35) [40].

The study showed that illiterate TB patients were found to be more than twice more likely to develop TB compared to patients who can at least read and write in local language and more levels (AOR = 2.15, with 95% confidence interval [CI]: 1.05, 4.40). Similar results were also reported in other studies, OR = 1.90, 95% CI: 1.24, 2.93) [48], OR = 1.90, 95% CI: 1.24, 2.93) [41] and OR = 2.42, 95% CI 1.09–5.37) [42].

Patients with household income of less than 1000 birrs per month were more than two times more likely to develop TB compared to those who had higher income (AOR = 2.2; 95% CI: 1.28, 3.78). This was consistent with other studies, OR = 4.12, 95% CI 2.53–6.71) [42], AOR = 1.1, CI = 0.34–0.47 [43], and also in India [44].

Smoking has also been identified as important risk factor for developing TB by four times (AOR = 4.43; 95% CI: 2.10, 9.3). This was well established fact in several studies, for instance, AOR = 3.90; 95% CI = 1.20–12.40) [39], OR = 2.12, 95% CI 1.20–3.74) [42] and statistically significant effects [45]. Smoking results in histological changes in the lower respiratory tract, including peri-bronchial inflammation, fibrosis, vascular intimal thickening, and destruction of alveoli. This leads to alterations in the epithelial function, such as reduced ciliary activity, decreased clearance of inhaled substances, and abnormal vascular and epithelial permeability, and in effect increasing the risk of developing pulmonary diseases such as TB. However the prevalence of smoking in this study is so low to explain any possible association.

BCG was found to be protective against TB reducing the risk by one-third (AOR = 0.34; 95% CI: 0.22, 0.54). This has be documented in other studies, where absence of BCG vaccination, as significant risk factors for LTBI [46] and that could facilitate disease transmission [47]. Those with BCG vaccination were less likely to have occupationally-acquired PTB than those without vaccination (OR = 0.86, 95% CI = 0.20–3.6 [48].

We acknowledge possibilities for residual confounders in this study. Those with high household income are likely to live in houses with multiple windows, better awareness about prevention and control, better access to health service, BCG vaccination, etc. A big scale researches with adequate sample size and sufficient representation of the diverse variables can help to filter out the relative effect of each factor.

## Conclusion

This case control study explored different socio-demographic factors and demonstrated some of the factors contributing to occurrence of TB disease. It showed that TB is more common among the most agile and economically active age group, males, low income segment of the society, overcrowded and poor standard of living.

Several other factors were also studied including risk associated with having a household members who had TB, smoking cigarettes, history of visiting a health facility, etc. In the final analysis the study identified seven key factors as standout risk factors for tuberculosis in this setting which are illiteracy, low household income, living in house without or single window, household member with TB and previous history of hospital admission. BCG scar was found to be protective factor.

Therefore it is imperative that the TB control effort need a strategy to address broader socio economic issues such as poverty, overcrowding, smoking as elements of the national response to control TB. Infection control at health care facilities level is an important intervention to prevent transmission of TB within the facilities in order to protect visitors, patients as well as health care providers, and need to be implemented across the health system from health posts to hospitals.

## Supporting information

S1 File (XLSX)

S2 File (ZIP)
