# Supplementary material for: Risk factors for tuberculosis: A case–control study in Addis Ababa, Ethiopia
Source: PLoS One. 2019 Apr 2;14(4):e0214235. doi: 10.1371/journal.pone.0214235 (PMC6445425; doi:10.1371/journal.pone.0214235)
Supplement: S2 File — (ZIP) [file pone.0214235.s002.zip › Questionnaire Plos one/English questionnaire-TB risk factor-cases.docx]

# ANNEX II: QUESTIONNAIRE FOR ASSESSMENT OF EPIDEMIOLOGIC DETERMINANTS OF TB DISEASE

**SECTION I: ASSESSMENT OF RISK FACTORS FOR TB- Questionnaire for cases**

**Instructions:**

**Please ask the following questions to respondents who full-fill the following criteria;**

- **Age greater than 15 years old**
- **New smear positive TB patients (cases)**

**After the individual have given written consent, please thank the respondent and ask each of the following questions and record the responses correctly.**

**Name of health facility:**

**Name of data collector:**

**Name of Supervisor:**

**Respondent’s unique identifier:**

**Date of interview**

| 1. **SOCIO-DEMOGRAPHIC DATA** | | | | |
| --- | --- | --- | --- | --- |
| **SN.** | **QUESTIONS** | **CHOICES** | **RESPONSE** | **SKIP** |
| 0101 | How old are you? | Write in years |  |  |
| 0102 | Sex | 1. M 2. F |  |  |
| 0103 | What is your marital status? | 1. Married 2. Unmarried 3. Widowed 4. Divorced |  |  |
| 0104 | What is your educational status? | 1. Illiterate 2. Read and write local language 3. Elementary (1-8) 4. Secondary school (9-12) 5. College or more |  |  |
| 0105 | What do you do for living? | 1. Health worker 2. Civil servant other than health worker: 3. Merchant 4. Student 5. Unemployed 6. Housewife 7. Other (specify) : |  |  |
| 1. **LIVING CONDITION** | | | |  |
| 0201 | Whom are you living with? | 1. Family members (parents, siblings, children, other relatives) 2. Alone 3. Congregate settings 4. Street life 5. Other (specify) : |  |  |
| 0202 | Have you ever lived in congregate settings? | 1. Yes 2. No |  |  |
| 0203 | What is the size of the family you are living with: | Write number of persons |  |  |
| 0204 | What is your average household monthly income? | Write amount in birrs |  |  |
| 0205 | How many rooms are there in your house? | Write number of rooms |  |  |
| 0206 | What is the volume of your house? | Write the size in m^3^ ***(assist to make reasonable estimate)*** |  |  |
| 0207 | How many windows does your house has? | 1. No window 2. One 3. Two 4. Three 5. More than three |  | To 0209 |
| 0208 | How often does the window at your house remain open during the day-time when people are in the room? | 1. Whole day 2. Half day 3. 2-3 hours 4. Never |  |  |
| 0209 | Was/is there any member of the family who had/has TB disease? | 1. Yes 2. No |  | To 0211 |
| 0210 | When did that person suffered from TB? | Write calendar year (EC): |  |  |
| 0211 | Was/Is there any person in work place who had/has TB? | 1. Yes 2. No |  | To 0301 |
| 0212 | If the answer to question 16 is yes, when did that person suffered from TB? | Write calendar year (EC): |  |  |
| 1. **PERSONAL LIFE STYLE** | | | |  |
| 0301 | Do you smoke cigarette? | 1. Yes 2. No |  | To 0303 |
| 0302 | If so, how many do you smoke per day? | 1. Less than 5 2. 6-10 3. 10-20 4. More than 20 |  |  |
| 0303 | Do you drink alcohol? | 1. Yes 2. No |  | To 0401 |
| 0304 | If so, how often? | 1. Daily 2. Twice per week 3. on average once per week 4. on average once per month 5. Rarely |  |  |
| 1. **CURRENT MEDICAL PROBLEM** | | | |  |
| 0401 | How long has it been since you started coughing? | ***Write duration in weeks*** |  |  |
| 0402 | Date of confirmation of diagnosis of TB? | ***Write*** *(* ***dd/mm/yy****)* | __/__/__ |  |
| 0403 | Date of starting Treatment | ***Write*** (**dd/mm/yy**) | __/__/__ |  |
| 1. **PAST MEDICAL HISTORY** | | | |  |
| 0501 | Do you have any past chronic illness? | 1. Yes 2. No |  | To 0503 |
| 0502 | If so, do you have any of the following? (***multiple answer possible***) | 1. Diabetes mellitus 2. Asthma 3. Chronic obstructive pulmonary diseases 4. Psychiatric problems 5. Other (Specify) |  |  |
| 0503 | Have you been vaccinated for BCG? (**Check left or right deltoid for BCG scar**) | 1. Yes 2. No |  |  |
| 0504 | Have you ever visited a health facility in the past 12 months? | 1. Yes 2. No |  | To 0508 |
| 0505 | What was the type of health facility you visited? | 1. Hospital 2. Health centre 3. Private clinic 4. Traditional healer 5. Other (specify) |  |  |
| 0506 | For what purpose did you visit that health facility? | 1. Seeking service 2. Visiting admitted patient 3. Escorting a patient 4. Working there 5. Other (specify) |  |  |
| 0507 | How many times did you visit these health facilities during the past 12 months? | (***write number of visits***) |  |  |
| 0508 | Have you even been admitted to a hospital? | 1. Yes 2. No |  | To 0601 |
| 0509 | When were you admitted | (***write the calendar year in EC***) |  |  |
| 0510 | How long were you admitted for? | (***write duration in days***) |  |  |
| 1. **PHYSICAL AND MEDICAL CONDITIONS** | | | |  |
| 0601 | Weight : | Write in Kgs |  |  |
| 0602 | Height: | Write in meters |  |  |
| 0603 | HIV test status ((only if indicated on card ) | 1. negative 2. positive 3. not tested 4. unknown 5. declined |  |  |
