# Supplementary material for: Risk factors for tuberculosis: A case–control study in Addis Ababa, Ethiopia
Source: PLoS One. 2019 Apr 2;14(4):e0214235. doi: 10.1371/journal.pone.0214235 (PMC6445425; doi:10.1371/journal.pone.0214235)
Supplement: S2 File — (ZIP) [file pone.0214235.s002.zip › Questionnaire Plos one/Questionnaire Amaharic -TB risk factors-controls.docx]

**የቲቢ በሽታን ኢፒደሚዮሎጂክ ሁኔታዎች ለማጥናት የሚደረግ ጥናት መጠይቅ (control)**

**ክፍል 1 ለቲቢ የሚያጋልጡ ሆኔታዎችን ስለማጥናት**

**መመሪያ፡**

የሚከተሉትን ጥያቄዎች ከመጠየቅዎ በፊት የተመረጡት መላሾች እነዚህን መስፈርቶች ማሟላታቸውን ያረጋጋጡ፣

- ፆታ እና ዕድሜአቸው ከ case ጋ*ር* ተመተሳሳይ የሆነ ( **ግፋ ቢል የአምስት ዐመት ልየነት)**
- **የ**ቲቢ በሽታ ምልክት የሌለው

መላሹ ፈቃደኝነቱን ካረጋገጠ በኋላ ምስጋና በማቅረብ የሚከተሉትን ጥያቄዎች በመጠየቅ መልሶቻቸውን በአግባቡ ይመዝግቡ፡፡

የጤና ጣቢያው ስም፡-

ለመላሹ የተሰጠው ምስጢራዊ ኮድ ***(የጤና ጣቢያው ስም፡ እና የመ ላሹ ተራ ቁጥር ከ አንድ ጀምሮ፤)***

የመረጃ ስብሰባው ስም፡-

መረጃ የተሰበሰበበት ቀን፡-

1. **ማህበራዊ መረጃ**

| **ተ.ቁጥር** | **ጥያቄ** | **ምርጫዎች** | **ማስታወሻ** |
| --- | --- | --- | --- |
| 0101 | ዕድሜዎ ስንት ነው? | ዐመት |  |
| 0102. | ፆታ | 1. ወ 🞏 2. ሴ 🞏 |  |
| 0103 | የጋብቻ ሁኔታዎ ምንድን ነው? | 1. ያገባ 2. ያላገባ 3. የትዳር አጋር የሞተበት 4. የተፋታ |  |
| 0104 | የትምህርት ደረጃዎ ምንድን ነው? | 1. ያልተማረ 2. ማንበብና መፃፍ የሚችል 3. መጀመሪየ ደረጃ ትምህርት የደረሱ /1-8/ 4. ሁለተኛ ደረጃ ምህርት የደረሰ /9-12/ 5. ኮሌጅ ወይም ከዛ በላይ የተማረ |  |
| 0105 | ስራዎ ምንድን ነው? | 1. የጤና ባለሙያ 2. የመንግስት ሠራተኛ /ከጤና ባለሙያ የተለየ/ 3. ነጋዴ 4. ተማሪ 5. ስራ የሌለው 6. የቤት እመቤት   7.ሌላ ካለ ይገለጽ |  |
| 1. **ያናናር ሁኔታ** | | | |
| 0201 | ከማን ጋር ነው የሚኖሩት? | 1. ከቤተሰብ ጋር /ከወላጅ፣ወንድም፣እህት፣ልጆች፣ዘመድ/ 2. ብቻውን የሚኖር 3. ከብዙ ሠዎች ጋር በአንድ ላይ መኖር /አዳሪ ት/ቤት፣ወህኒ ቤት/ 4. የጐዳና ተዳዳሪ 5. ሌላ ካለ ይገለፅ |  |
| 0202 | በተፋፈገ ሁኔታ ከብዙ ሠዎች ጋር አብረው ኖረው ያውቀሉ? | 1. አዎ 2. የለም |  |
| 0203 | አብረዎት የሚኖሩ የቤተሰብ አባላት ብዛት ስንት ነው? | _____________ሠዎች  **(የአባላቱን ብዛት በቁጥር ፃፍ**) |  |
| 0204 | የቤተሰብዎ አማካይ ወራዎ ገቢ ስንት ነው? | 1. ከ1000 ብር በታች 2. ከ1000-2000 ብር 3. ከ2000-3000 ብር 4. ከ3000 ብር በላይ |  |
| 0205 | በቤትዎ ውስጥ ስንት ክፍሎች አሉ? | ክፍሎች |  |
| 0206 | የቤትዎ ስፋት ስንት በስንት ነው? | ሜትር በ ሜትር |  |
| 0207 | ቤትዎ ሰንት መስኮቶች አሉት? | 1. ምንም የለውም 2. አንድ 3. ሁለት 4. ሶስት 5. ከሶስት በላይ | ወደ 0209 |
| 0208 | የቤትዎ መስኮቶች በቀን ውስጥ ለምን ያክል ጊዜ ክፍት ሆነው ይቆያሉ? | 1. ሙሉ ቀን 2. ግማሽ ቀን 3. ከ2-3 ሠዓታት 4. ተከፍተው አያውቁም |  |
| 0209 | ከቤተሰቡ አባላት መካከል በቲቢ በሽታ የተያዘ ሠው ነበረ? | 1. አዎ 2. የለም | ወደ 0210 |
| 0210 | በቲቢ የተያዘ ሰው ከነበረ፣ መቼ ነበር የታመመው? | ዓመተ ምህረቱን ፃፍ፡ |  |
| 0211 | በሰራ ቦታዎ በቲቢ በሽታ የተያዘ ሠው ነበረ? | 1. አዎ 2. የለም | ወደ 0301 |
| 0212 | መልስዎ አዎ ከሆነ፣ መቼ ነበር የታመመው? | ዓመተ ምህረቱን ፃፍ |  |
| **3. የግል ባህሪያት** | | | |
| 0301 | ሲጋራ ያጨሳሉ? | 1. አዎ 2. የለም | ወደ 0303 |
| 0302 | የሚያጨሱ ከሆነ በቀን እስከ ስንት ሲጋራ ያጨሳሉ? | 1. ከ5 በታች 2. ከ6-10 3. ከ 10-15 4. ከ 15 በላይ |  |
| 0303 | መጠጥ ይጠጣሉ? | 1. አዎ 2. የለም | ወደ 0401 |
| 0304 | የሚጠጡ ከሆነ በየስንት ጊዜው ይጠጣሉ? | 1. በየዕለቱ 2. በሳምንት ሁለት ቀናት 3. በሳምንት አንድ ቀን 4. በወር አንዴ 5. አልፎ አልፎ |  |
| **5. የበፊት የጤና ሁኔታ** | | | |
| 0501 | ለብዙ ጊዜ የቆየ በሽታ አለብዎ? | 1. አዎ 2. የለም | ወደ 0503 |
| 0502 | ለብዙ ጊዜ የቆየ በሽታ ካለብዎ፣ ከተዘረዘሩት የለብዎትን ይናገሩ ***(የተጠቀሱትን ሁሉ ክበብ)*** | 1. የስኳር በሽታ 2. አስም 3. ለረጅም ጊዜ የቆየ የሳንባ በሽታ /COPD/ 4. የአዕምሮ በሽታ 5. ሌላ ካለ ይገለፅ |  |
| 0503 | በልጅነትዎ የቲቢ ክትባት ተከትበዋል?  **(ለማረጋገጥ በግራ ወይም በቀኝ እጅ ላይ ጠባሳ መኖሩን ይመለከቱ)** | 1. አዎ 2. የለም |  |
| 0504 | ባለፋት 12 ወራት ውስጥ ወደ ጤና ተቋም ሄደው ነበር? | 1. አዎ 2. የለም | ወደ 0508 |
| 0505 | ምን አይነት ጤና ተቋም ነው ሄደው የነበረው?  ***(የተጠቀሱትን ሁሉ ክበብ)*** | 1. ሆስፒታል 2. ጤና ጣቢያ 3. የግል ተቋም 4. የባህል ህክምና 5. ሌላ ካለ ይገለፅ |  |
| 0506 | ወደ ጤና ተቋሙ የሄድት ለምን ነበር? | 1. ለመታከም 2. የታመመ በሽተኛን ለመጠየቅ 3. በሽተኛን ለማሳከም 4. እዛው ስለምሠራ 5. ሌላ ካለ ይገለፅ |  |
| 0507 | ባለፋት 12 ወራት ውስጥ ወደ ጤና ተቋማት ስንት ጊዜ ሄዱ? | 1. አንድ ጊዜ 2. ከሁለት እስከ አምስት ጊዜ 3. ከአምስት ጊዜ በላይ |  |
| 0508 | ታመው ሆስፒታል ተኝተው ያውቃሉ? | 1. አዎ 2. የለም | ወደ 0601 |
| 0509 | መቼ ነበር ተኝተው የታከሙት? | ዓመተ ምህረቱን ፃፍ |  |
| 0510 | ለስንት ጊዜ ነበር ተኝተው የታከሙት? | (ጊዜውን በቀናት ግለፅ) |  |
| **6. አካላዊ እና የጤና ሁኔታ** | | | |
| 0601 | ክብደት | ኪ.ግ |  |
| 0602 | ቁመት፡ | ሜትር |  |
| 0603 | የኤች አይ ቪ ምርመራ ውጤት /የታወቀ ከሆነ/ | 1. ኔጌቲቭ 2. ፐዘቲቭ 3. አልተመረመሩም 4. አልታወቀም 5. ለመመርመር ፍቃደኛ አይደሉም |  |
